# Fabry_CEP: a tool to identify Fabry mutations responsive to pharmacological chaperones

**DOI:** 10.1186/1750-1172-8-111

**Published:** 2013-07-24

**Authors:** Marco Cammisa, Antonella Correra, Giuseppina Andreotti, Maria Vittoria Cubellis

**Affiliations:** 1 Dipartimento di Biologia, Università Federico II, Napoli, Italy; 2 Istituto di Chimica Biomolecolare –CNR, Pozzuoli, Italy; 3 Istituto di Biostrutture e Bioimmagini-CNR, Napoli, Italy

**Keywords:** Fabry disease, Pharmacological chaperone, Web-application, Therapy

## Abstract

Fabry_CEP is a user-friendly web-application designed to help clinicians Choose Eligible Patients for the therapy with pharmacological chaperones. It provides a database and a predictive tool to evaluate the responsiveness of lysosomal alpha-galactosidase mutants to a small molecule drug, namely 1-Deoxy-galactonojirimycin. The user can introduce any missense/nonsense mutation in the coding sequence, learn whether it is has been tested and gain access to appropriate reference literature. In the absence of experimental data structural, functional and evolutionary analysis provides a prediction and the probability that a given mutation is responsive to the drug.

## Letter to the Editor

Pharmacological chaperones (PC) offer a promising therapeutic strategy for Fabry disease (FD). Most experiments have focused on 1-deoxy-galactonojirimycin, also known as DGJ, migalastat-hydrochloride or AT1001. The decision to use it in therapy has to be taken on a case-by-case basis after precise genotyping because DGJ is not effective on all alpha-galactosidase (AGAL) mutants. Thirteen out of 450 missense/nonsense mutations [1] have been tested in clinical trials [2,3] and 130 in cells (for a review [4]). Fabry_CEP [5] helps finding experimental data for a given mutation. The reference sequences for wild-type (EMBL: X05790 or UniProt: AGAL_HUMAN for the open reading frame or for amino-acid sequence, respectively) are provided and the substitute nucleotide or amino acid can be selected. If experimental data are available, the user will get an answer taken from the literature to whether the mutation is responsive to DGJ. A non-sense mutation generates a truncated protein which is not amenable for PC and Fabry_CEP [5] provides a negative conclusion. Disruption of a disulphide bond or occurrence into the active site (as defined in [6]) are the only structural features sufficient, but not necessary, to prevent responsiveness to DGJ. When these conditions occur, Fabry_CEP [5] gives a negative outcome. The structure of wild-type AGAL is displayed with the residues involved in disulphide bridges highlighted in yellow and those of the active site in blue. Upon selection, the site affected by the mutation is shown on a space-filling model and colored in yellow or in blue, if it occurs at disulphide bridge sites or in the active site, in magenta otherwise. Recently, it was proposed that mutations promoting protein aggregation do not respond to PC [7]. We ran the program TANGO [8] and we found that a small minority, twelve out of 130 mutations experimentally tested Fabry, is predicted to promote aggregation. Indeed mutations with high TANGO score tend to be non responsive, but the condition is neither necessary nor sufficient to prevent responsiveness.

**Figure 1:**
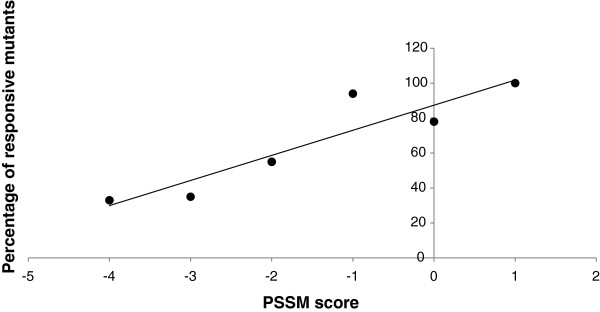
Correlation of responsiveness to pharmacological chaperones with scores assigned with a Position Specific Substitution Matrix (PSSM).

If a mutation does not occur in the active site and does not disrupt disulphide bonds, Fabry_CEP [5] uses a Position Specific Substitution Matrix (PSSM) score [9]. In brief the method considers the degree of conservation and the type of amino acid introduced at the site of the mutation. For these mutations an outcome can be given only in probabilistic terms. We re-evaluated the correlation between PSSM scores and the percentage of responsive mutations tested experimentally for this subset of cases (113). We obtained a Pearson correlation coefficient r = 0.92 with two-tailed p-value 0.01 (Figure 1).

AGAL contains a signal peptide of 31 aa. Although a PSSM score might be calculated also for mutations occurring in this peptide, we prefer to not give a conclusion because there are no experimental data in this region to benchmark the prediction.

**Figure 2:**
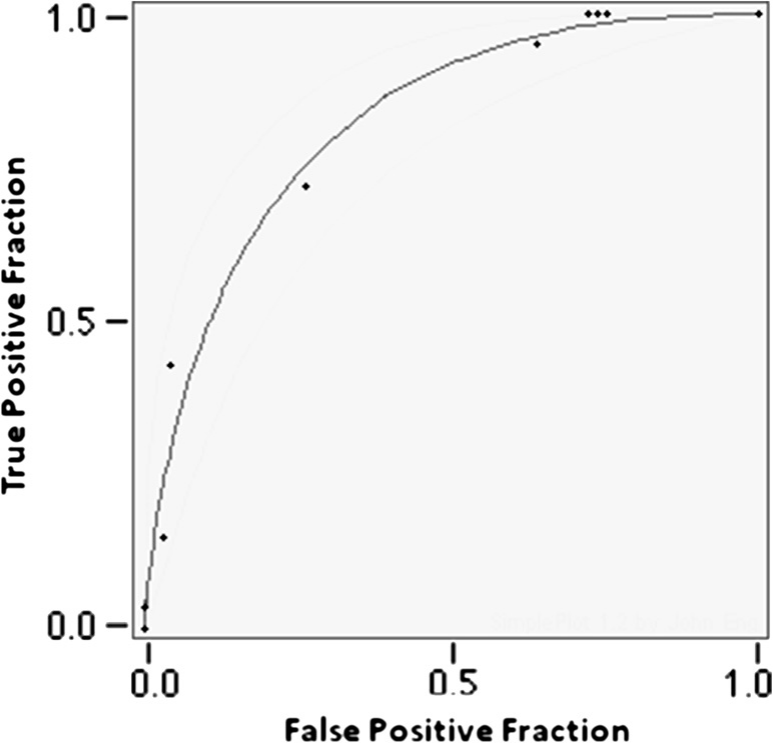
Performance of Fabry_CEP illustrated by receiver operating characteristic (ROC) curve.

The complete test-set used to build Fabry_CEP [5] falls in nine discrete rating categories. We built the receiver operating characteristic (ROC) curve shown in Figure 2 and measured empiric and fitted ROC Areas, 0.807 and 0.825 respectively.

**Figure 3:**
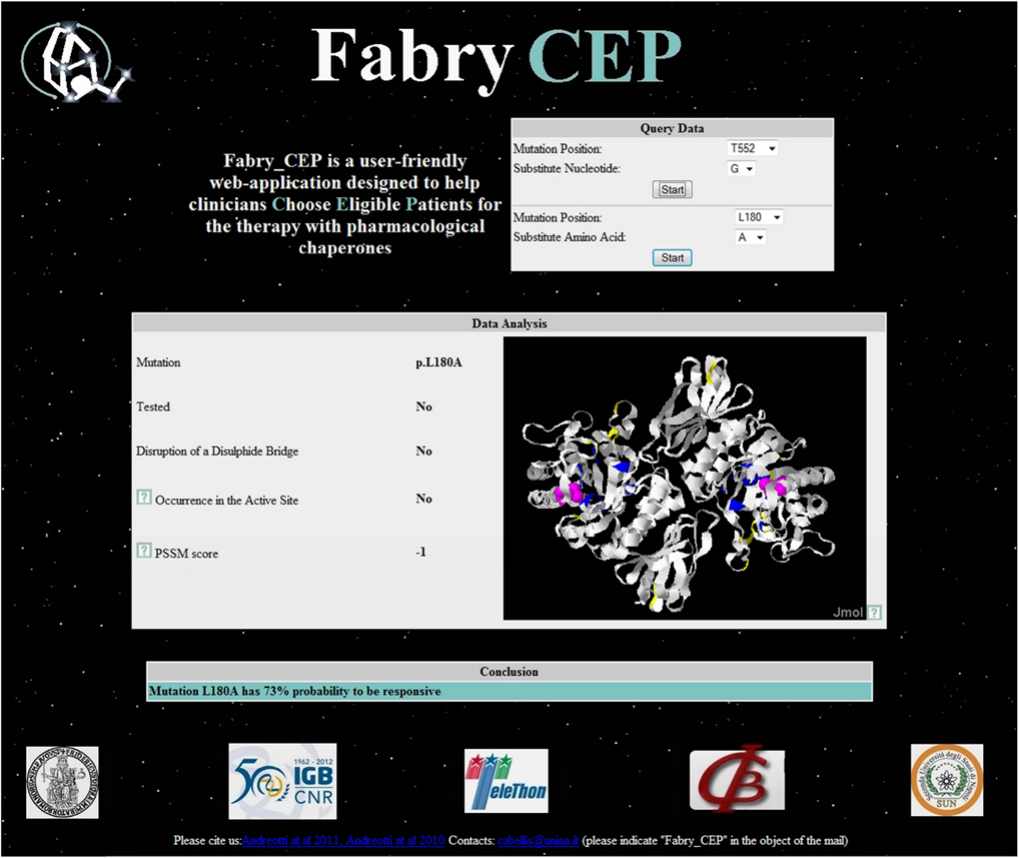
Fabry_CEP interface.

Figure 3 illustrates a representative case analyzed by Fabry_CEP [5] with a upper section for the query, a middle section for the intermediate results on which the conclusion is drawn and a lower section with the section showing the final outcome.

Private mutations are common in Fabry disease and missense mutations are very common, so it is expected that this tool may progressively gain more importance in the future. However a word of caution is needed. Fabry_CEP [5] can be used as a predictive tool, but its clinical applicability is limited and individual decisions on whether to start or not DGJ should not be based solely on this tool.

## Abbreviations

PC: Pharmacological chaperones; FD: Fabry disease; AGAL: Alpha-galactosidase; PSSM: Position specific substitution matrix.

## Competing interests

MVC was a consultant for Shire HGT.

## Authors’ contributions

GA and MVC designed the study and wrote the paper. MC and AC built the web-application. All authors read and approved the final manuscript.
